# Advances in Bone Biology

**DOI:** 10.3390/ijms25136892

**Published:** 2024-06-23

**Authors:** Maria Rosa Iaquinta, Monica Montesi, Elisa Mazzoni

**Affiliations:** 1 Department of Medical Sciences, University of Ferrara, 64/b, Fossato di Mortara Street, 44121 Ferrara, Italy; qntmrs@unife.it; 2 National Research Council of Italy—Institute of Science, Technology and Sustainability for Ceramics CNR—ISSMC, Via Granarolo 64, 48018 Faenza, Italy; monica.montesi@issmc.cnr.it; 3 Department of Chemical, Pharmaceutical and Agricultural Sciences, University of Ferrara, 48018 Ferrara, Italy

Bone is a unique type of mineralised connective tissue that can support and protect soft tissues, contain bone marrow, and allow movement. Although bone may appear to be a static structure, it is a highly active organ that is constantly changing throughout life. Bone remodelling is a complex process involving the coordinated actions of osteoblasts and osteoclasts to maintain bone homeostasis. The process of bone remodelling is regulated through interactions between osteoblast-mediated bone production and osteoclast-mediated bone resorption [1]. Mesenchymal stem cells (MSCs) are the source of osteoblasts, which comprise 4–6% of all the resident cells in bone. Their main job is to produce bone matrix by secreting proteoglycans and type I collagen, which is followed by mineralisation. Osteoclasts derive from a distinct cell source; they are derived from haematological mononuclear cells that undergo differentiation and activation to create massive, multinucleated, highly motile cells that perform bone resorption. Osteoclasts are specialised cells that grow and adhere to the bone matrix. They are produced from the monocyte/macrophage haematopoietic lineage. In an extracellular compartment, they produce lytic enzymes and acid to break down the matrix. Understanding the mechanisms of osteoclastogenesis and activation of bone resorption, as well as the effects of hormone signals on bone mass and structure, has become possible with the discovery of the nuclear factor kappa B receptor activator (RANK) signalling pathway in the osteoclast. When the homeostatic balance between these two cell types is dysregulated, this can contribute to abnormal bone remodelling, resulting in a loss of bone mass, as is observed in degenerative bone metabolic diseases, e.g., osteoporosis (OP). In this context, it is important to highlight that approximately 20 million patients/year suffer from bone tissue loss due to trauma or diseases [2].

This Special Issue aims to highlight the scientific progress made in the biology related to bone tissue and comprises seven articles, including five original research articles, one communication, and one review. Specifically, Xueqin Gao et al. [3] emphasised the need for identifying new factors to prevent or treat bone loss due to OP and age-related diseases. These authors examined Murphy Roths Large/lymphoproliferation (lpr) strain (MRL/MpJ) mice, also called “super healer mice”, for resistance to age-related and long-term ovariectomy-induced bone loss to identify putative protective factors for bone repair and regeneration. MRL/MpJ mice have shown an enhanced capacity for healing several musculoskeletal tissue injuries, such as articular cartilage/tendinous lesions, skin and ear wounds, and nerve injuries. During ageing and long-term ovariectomy, MRL/MpJ mice maintained more bone mass and better bone microarchitecture than wild-type (WT) mice. The preservation of larger osterix (OSX^+^) osteoprogenitor cell pools, increased phosphorylated small mothers against decapentaplegic 5 (pSMAD5) signalling pathway activation, more proliferating cell nuclear antigen (PCNA^+^) cells, and fewer osteoclasts were all associated with the resistance to bone loss shown in MRL/MpJ animals. In MRL/MpJ mice, compared to WT mice, lower levels of RANKL and DKK1, together with greater serum levels of insulin-like growth factor (IGF1) and osteoprotegerin (OPG), may also help to maintain higher bone microarchitecture over age and less severe bone loss following long-term ovariectomy.

It is well known that OPG, a novel member of the tumour necrosis factor (TNF) receptor superfamily, binds to RANK in a competitive manner with nuclear factor kappa B receptor activator ligand (RANKL) to decrease osteoclast development and activity [4]. In addition, OPG can induce apoptosis in mature osteoclasts in vitro, preserving bone structure. Pyroptosis is a recently discovered form of cell death that is distinct from apoptosis. In comparison to apoptosis, pyroptosis is an unusual type of cell death that was identified in the 1980s and is characterised by the rupture of cell membranes and the release of inflammatory chemicals. Jiaqiao Zhu et al. [5] reported that OPG can induce osteoclast pyroptosis, and its molecular mechanism is related to the expression levels of ASC, NLRP3, caspase-1, and GSDMD, which were included in the classical pathway of pyroptosis, and offered new suggestions for treating clinical bone metabolic disorders [5]. 

Extracellular signal-regulated kinases (ERKs) are also known to play significant roles in regulating osteoclast growth and differentiation. Eun-Bi Choi et al. [6] reported that ERK inhibitors, i.e., PD98059 and U0126, increased RANKL-induced osteoclast differentiation in RAW 264.7 cells, implying a negative role in osteoclast differentiation. The ERK inhibitors led to an increase in the phosphorylation of adenosine 5′-monophosphate-activated protein kinase (AMPK) but not the activation of p38 MAPK, Lyn, and mTOR. In addition, while the ERK inhibition increased the expression of the RANKL receptor RANK, it decreased the expression of negative mediators of osteoclast differentiation, including interferon regulatory factor-8, B-cell lymphoma 6, and interferon-γ. Their results provide important information for drug development employing ERK inhibitors for the treatment of osteoclast-related diseases.

Parichart Toejing and colleagues [7] investigated the skeletal phenotype of transgenic mice expressing constitutively active TGF-β receptor type I under the control of Mx1-Cre (Mx1;TβRI^CA^ mice). Their histomorphometric analysis confirmed a decrease in cancellous bone volume due to increased osteoclast number and decreased osteoblast number. Primary osteoblasts showed decreased ALP and mineralisation, together with an increase in osteoclast differentiation. In Mx1; TβRI^CA^ mice, it has been observed that there is an increase in osteoclast-related genes and a reduction in osteoblast-related genes.

In their review, Qiuyue Qin et al. [8] explored the hypoxia-inducible factors signalling osteogenesis and skeletal repair. Oxygen is a signalling molecule that controls cell-to-cell communication, motility, differentiation, metabolism, and cellular survival. Under physiological and pathological conditions, hypoxia has a specific role in maintaining bone homeostasis. Nonetheless, hypoxia also has a role in aiding in fracture healing. Hypoxia-inducible transcription factors (HIFs) are key players in homeostasis regulation; they are necessary for the control of osteogenesis and skeletal repair and can enable cells to endure in low-oxygen environments. Factors HIF-1 and HIF-2 are fundamental in signalling pathways linked to skeletal repair and bone development, along with the molecular mechanisms governing the regulation of subsequent growth factors and protein molecules like vascular endothelial growth factor (VEGF) and EPO. The transcription factors HIF-1 and HIF-2 and their target genes regulate osteogenic processes in response to hypoxia systematically. This established the new concept that bone growth and regeneration are regulated by the concerted actions of multiple types of skeletal stem cells, which reside in spatiotemporally distinct niches. 

One of the fundamental rules regulating the physiology of bone remodelling is epigenetics. DNA methylation, histone modifications, and non-coding RNAs are the key epigenetic modifications that control stable transcriptional programmes without establishing particular heritable changes. DNA methylation in CpG-rich promoters of the gene is primarily correlated with gene silencing, and histone modifications are associated with transcriptional activation/inactivation. N6-methyladenosine (m^6^A) has been a critical regulator of gene expression in a wide range of biological activities in recent years. 

A recent study was designed to investigate the roles of m^6^A demethylases in dental follicle stem cells. Linwei Zheng et al. [9] provide a source of data on miRNA and mRNA that were differently expressed following m^6^A demethylase overexpression in dental follicle stem cells (DFSCs). M^6^A demethylase inhibits osteogenesis in DFSCs via the miR-7974/FK506-binding protein 15 (FKBP15) pathway. The effects of FK506-binding protein 15 (FKBP15), miR-7974, and m^6^A demethylase on DFSC osteogenesis that are not dependent on RUNX2. Additionally, it has been discovered that the FTO/miR-7974/FKBP15 axis influences the structure of the actin cytoskeleton in DFSCs.

To characterise resident synovial progenitor populations, Katarina Barbarić Starčević et al. analysed the cellular composition of synovial tissue from 11 patients with rheumatoid arthritis (RA) and 10 control patients harvested during planned surgeries to characterise resident synovial progenitor populations [10]. Briefly, RA is a chronic, autoimmune condition marked by irreversible joint degeneration and joint inflammation. In addition to increased resorption, lower bone production results from mesenchymal lineage-derived osteoblasts’ inhibited differentiation and function in an inflammatory environment. Mesenchymal lineage progenitor cells are seen in synovia. These cells can develop into bone and cartilage and are distinguished by a variety of surface markers. These authors revealed that the proportions of lymphocytes (CD3^+^ and CD19^+^) and myeloid (CD11b^+^ and CD14^+^) cells were higher in synovial tissue from RA patients compared to controls. Among non-hematopoietic cells (e.g., CD45^−^ CD31^−^ CD235a^−^ cells), there was a decrease in the proportion of CD200^+^ CD105^−^ cells and an increase in the proportion of CD200^−^ CD105^+^ cells in synovial tissue from the RA patients in comparison to the controls.

In conclusion, the collective findings presented in this Special Issue offer a deep insight into the multifaceted landscape of bone repair. These studies provide new information on the regulatory mechanisms of bone regeneration. Moving forward, continued interdisciplinary research and collaboration will be essential to translating these discoveries into tangible benefits for patients, ultimately paving the way towards more effective prevention and treatment of joint and bone-related diseases.
